# Feasibility of Transcatheter Caval Valve Implantation to Improve **Sleep**-Disordered Breathing in Patients With Severe Tricuspid Regurgitation—A Pilot Study

**DOI:** 10.3389/fcvm.2021.673164

**Published:** 2021-07-19

**Authors:** Youmeng Wang, Roberto Fernandes Branco, Andrea Fietzeck, Thomas Penzel, Christoph Schöbel

**Affiliations:** ^1^Sleep Medicine Center, Charité-Universitätsmedizin, Berlin, Germany; ^2^Universitätsmedizin Essen, Ruhrlandklinik—Westdeutsches Lungenzentrum am Universitätsklinikum Essen GmbH, Essen, Germany

**Keywords:** sleep-disordered breathing, tricuspid regurgitation, right heart failure, transcatheter caval valve implantation, ApneaLink

## Abstract

Transcatheter caval valve implantation (CAVI) has been evaluated as a treatment option for inoperable patients with severe symptomatic tricuspid regurgitation (TR). We studied the effect of CAVI on sleep disorder breathing (SDB) in patients with right heart failure and TR. Twenty right heart failure patients with severe symptomatic TR who underwent portable monitoring of SDB (ApneaLink), echocardiography, cardiopulmonary exercise (CPET), and laboratory testing were enrolled. This was a single-center, nonblinded study. There were no significant changes in sleep variables, echocardiographic parameters, laboratory results, lung function, and CPET after CAVI. In conclusion, these data suggest that CAVI may not have an effect on SDB; however, additional follow-up fully powered studies with appropriate statistical analyses are needed.

## Introduction

Chronic heart failure (CHF) is an increasing health problem affecting more than 25 million people worldwide. The prevalence of heart failure in developed countries is about 1–2% in general and over 10% in patients above 70 years (1). Despite improvement in treatment, the prognosis of CHF is still poor, and more than 50% of hospitalized patients with CHF die within 5 years. Patients with CHF commonly suffer from breathing disorders during sleep (2, 3). Sleep disorder breathing (SDB) is a highly prevalent comorbidity in CHF patients, which has adverse effects on the prognosis of CHF. The presence of breathing disorders during sleep in patients with CHF is associated with increased risk of cardiovascular disease and mortality (4, 5). There are two different types of SDB in patients with HF: obstructive sleep apnea (OSA) and central sleep apnea (CSA) (6, 7).

Severe tricuspid regurgitation (TR) is a complex condition of the right ventricle (RV) and tricuspid valve apparatus and is frequently associated with symptomatic heart failure (8). The etiology of TR can be divided into primary and secondary causes. Primary TR may be caused by congenital, traumatic, rheumatic, and endomyocardial fibrosis. In these patients, left heart diseases could lead to chronic pressure overload of the RV, which eventually resulted in progressive RV expansion and functional TR. In patients with severe TR, medical therapy restricted to diuretics and heart failure medication is frequently infective, and surgical repair is associated with a high risk of morbidity and mortality (9, 10). In addition, neither one of these treatment options has demonstrated beneficial long-term effects. Therefore, multiple innovative interventional treatment concepts to replace or repair tricuspid valve function are currently under investigation.

CAVI has been suggested as one of these interventional concepts. In the pathological cascade of tricuspid valve disease, CAVI aims at the caval backflow that occurs at a late stage of severe TR (11). One previous study showed a decrease in the apnea–hypopnea index (AHI) after heart transplantation and medical treatment in a population of CSA patients with congestive heart failure group (*n* = 13) (12). Another study showed that one 64-year-old male patient with CSA improved his sleep, daytime hypersommolence, dyspnea, and fatigue after mitral valve transplantation (13). There has only been one randomized controlled trial that showed that transcatheter aortic valve replacement in patients with CHF and TR did not show positive effects on SDB (8, 10). The aim of our study was to examine whether CAVI has an effect on SDB in patients with right heart failure and TR.

## Materials and Methods

### Study Design and Collection of Data

**Figure 1:**
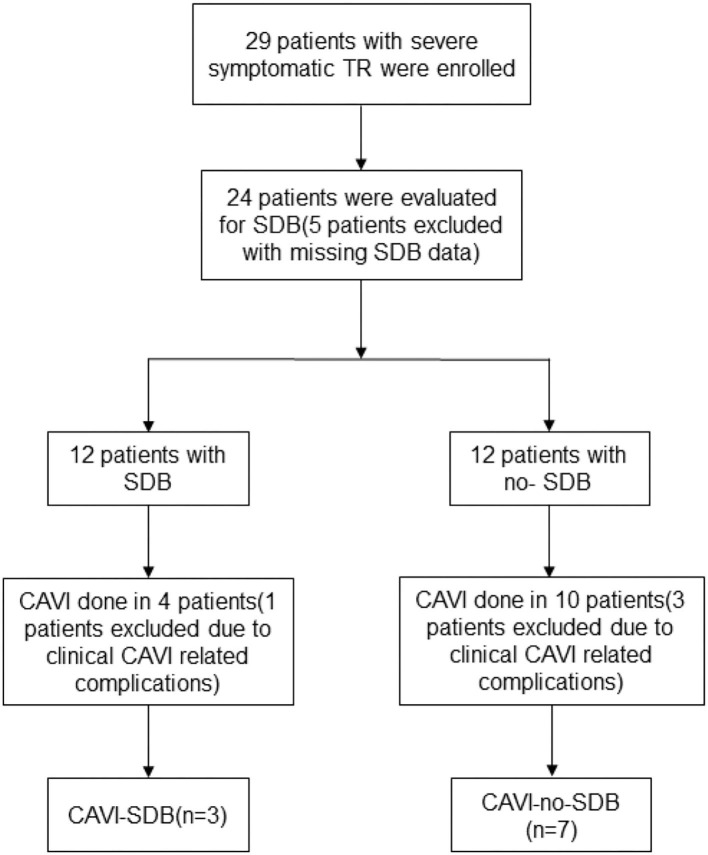
Patient selection and flow.

Our investigation was added on top of a previous study (11). This was a single-center, nonblinded study. Between January 2015 and November 2019, 29 consecutive right heart failure patients with severe symptomatic TR were divided into the CAVI group (*n* = 14) and the control group (*n* = 15), treated with optimal medical therapy (OMT) alone. The CAVI procedures were successful in all patients. An indicator of success was that CAVI resulted in the full reduction of reverse caval flow as confirmed by a significant reduction in the inferior vena cava (IVC) v-wave in all patients; this is already known in a previous study (9). Four major complications in the CAVI group that occurred within 48 h after implantation and resulted in open-heart surgery (two cases of cardiac tamponade secondary to stent migration and two valve dislocations) were excluded. After the fourth major complication, recruitment was stopped for safety. Five patients in the control group did not receive portable monitoring results and were excluded from this study. Patients with severe symptomatic TR were screened for SDB using a three-channel screening system (ApneaLink, Resmed). Data were collected at baseline and at 1-month follow-up from medical records. Portable monitoring was performed in 20 patients for SDB assessment. To diagnose SDB, AHI had to be 10/h or above. According to the value of the AHI, we divided the patients into four groups: control-SDB (*n* = 8), CAVI-SDB (*n* = 3), CAVI-no-SDB (*n* = 7), and control-no-SDB (*n* = 2) (Figure 1). All patients provided informed consent, and treatment was performed after the approval of the local ethics committee (Landesamt fur Gesundheit und Soziales Berlin, Germany).

### Patient Screening and Follow-Up

The inclusion criteria were the same as those defined by a previous study (11): (1) TR severity ≥ severe; (2) New York Heart Association (NYHA) functional class II or greater despite the established OMT; (3) age ≥ 50 years; and (4) high surgical risk. This study required that cardiac surgeons, interventional and non-interventional cardiologists, anesthesiologists, and imaging experts completed the evaluation and acceptance of CAVI patients. Routine preoperative examinations include transthoracic echocardiography, cardiopulmonary exercise, and laboratory examinations.

Exclusion criteria were as follows: (1) IVC diameter > 31 mm; (2) ongoing treatment of SDB; (3) inability to undergo portable monitoring; (4) serum creatinine concentration > 3.0 mg/dl; (5) patients for whom informed consent cannot be obtained; (6) chronic kidney disease undergoing regular dialysis; and (7) left ventricular ejection fraction < 30%. We offered CAVI to all severe symptomatic TR patients with SDB who met the inclusion criteria (11).

### Portable Monitoring

ApneaLink (Resmed) is a three-channel, portable device that uses a nasal pressure transducer to derive the AHI, flow limitation, and snoring, in addition to monitoring oxygen saturation during sleep. The oxygen desaturation index (ODI) was measured with the AL during the simultaneous study. The AL device operates on battery power, with a sampling rate of 100 Hz, and has a 16-bit signal processor. The internal memory storage is 15 MB, which allows ~10 h of data collection. The software analyzes the data generated by the flow signal, whereas full disclosure of data is available for review and rescoring by the clinician. AHI ≥ 10/h was defined as SDB in this study.

### CAVI Procedure

All implantations were performed through transfemoral venous access under local anesthesia and transthoracic echocardiography. After preparing the landing area by implanting a self-expanding stent (Sinus-XL, Optimed, Ettlingen, Germany) to facilitate valve fixation, the Sapien XT transcatheter valve (Edwards Lifesciences, Irvine, CA) was implanted in the IVC at the level of the diaphragm and protruding ≈5 mm into the right atrium (RA) (11).

### Laboratory Testing and Echocardiography

Blood sample collection: a nurse collected 2 ml venous blood, injected it into an anticoagulation tube, and fully blended it for later use. A biochemical auto-analyzer was applied to examine the eGFR. All patients underwent our standard 2-D and 3-D transthoracic and transesophageal echocardiography. Echocardiographic parameters included LVEF, RV-FAC, TAPSE, and the tissue Doppler *E*/*E*′ ratio. All recordings were performed on ultrasound systems.

### Lung Function and Cardiopulmonary Exercise Testing

Spirometry (FEV1, FVC; FEV1/FVC ratio), measurement of static lung volumes (total lung capacity (TLC) by body box plethysmography), and measurement of diffusing capacity of the lung for carbon monoxide (DLCO) by the single-breath technique were performed (Vmax22, SensorMedics, Yorba Linda, CA, USA) with the patient in the seated position. Testing protocols adhered to guidelines for calibration and testing recommended by the ATS/ERS standards. All patients performed a symptom-limited CPET by using a cycle ergometer protocol, which is cycling with a pedal speed of 60 rpm, starting at a workload of 20 W, followed by a stepwise 20-W increment every 2 min until exhaustion. AT was identified through a V-slope analysis of VO_2_ and CO_2_ production (VCO_2_), and it was confirmed through the specific behavior of the ventilatory equivalents of O_2_ (VE/VO_2_) and CO_2_ (VE/VCO_2_), as well as through the end-tidal O_2_ and CO_2_ pressure. The relation between VE and VCO_2_ was analyzed as the slope (VE/VCO_2_ slope).

### Statistical Analysis

Descriptive statistics (means and standard deviations) were used to describe baseline characteristics. Categorical variables are expressed as numbers (*n*) and percentages (%). Our data follow a normal distribution and were analyzed using a paired *t*-test and independent sample *t*-test for within-group and between-group comparisons, respectively. A value of *p* < 0.05 means statistically significant. All statistical data were performed using SPSS version 25.0 (IBM, Armonk, NY, USA).

## Results

The general characteristics of the participants at baseline are presented in Table 1. No significant differences in sex, age, body mass index (BMI), ejection fraction, NYHA functional class, and medications were observed between groups. All patients were taking loop diuretics, and they all had severe symptomatic TR. There were no changes in medication during the study.

**Table 1:** Baseline clinical data comparisons.

**Characteristic**	**CAVI-SDB (*n* = 3)**	**CAVI-no-SDB (*n* = 7)**	***P-*value**
Age, years	81 ± 3	69 ± 8	0.057
Female	2 (66.7%)	6 (85.7%)	1.000
Male	1 (33.3%)	1 (14.3%)	
BMI, kg/m^2^	29 ± 7	25 ± 4	0.233
**NYHA**			1.000
II	0 (0%)	0 (0%)	
III	3 (100%)	7 (100%)	
IV	0 (0%)	0 (0%)	
PAP mean, mmHg	26 ± 5	29 ± 18	0.814
PCWP mean, mmHg	16 ± 6	16 ± 9	0.975
Arterial hypertension	3 (100%)	7 (100%)	–
Nicotine abuse	1 (33.3%)	1 (14.3%)	1.000
COPD	1 (33.3%)	4 (57.1%)	1.000
GFR, ml/min	35 ± 16	49 ± 23	0.365
**Diabetes mellitus**			1.000
No diabetes	2 (66.7%)	5 (71.4%)	
Type 1	0 (0%)	1 (14.3%)	
Type 2	1 (33.3%)	1 (14.3%)	
LVEF, %	54 ± 10	61 ± 2	0.360
**Medication**
Beta blocker	3 (100%)	7 (100%)	–
Loop diuretics	3 (100%)	7 (100%)	–
Aldosterone antagonist	1 (33.3%)	3 (42.9%)	1.000
Statin	2 (66.7%)	3 (42.9%)	1.000
ACE	2 (66.7%)	6 (85.7%)	1.000
Antiplatelet	2 (66.7%)	3 (42.9%)	1.000
Oral anticoagulant	2 (66.7%)	1 (14.3%)	0.183
Calcium antagonist	1 (33.3%)	2 (28.6%)	1.000
Antiarrhythmic	0 (0%)	0 (0%)	–

*BMI, body mass index; ACE, angiotensin-converting enzyme; NYHA, New York Heart Association; COPD, Chronic Obstructive Pulmonary Disease; PAP, Pulmonary Artery Pressure; PCWP, Pulmonary Capillary Wedge Pressure; GFR, Glomerular filtration rate. Data were presented as mean ± SD or n (%). None of the variables is statistically significant*.

Comparisons between CAVI -SDB and CAVI-no-SDB groups are displayed in Table 2. There were no significant changes of all parameters before and after treatment in both groups (*p* > 0.05). By design, significant differences of tissue Doppler E/E, FVC % pred, FEV1, and FEV1 % pred were observed before the treatment between groups (*p* < 0.05). There were significant differences of LVEF, RVFAC, AHI, and ODI after the treatment between groups (*p* < 0.05).

**Table 2:** Comparisons between CAVI-SDB and CAVI-no-SDB group.

**Variables**	**CAVI-SDB**			**CAVI-no-SDB**				
	**Pre**	**Post**	***P***	**Pre**	**Post**	***P***	P^*^	***P*^**#**^**
LVEF, %	54 ± 10	49 ± 9	0.383	61 ± 2	63 ± 6	0.321	0.360	**0.021**
RVFAC, %	12 ± 0	30 ± 2	–	44 ± 13	48 ± 10	0.211	0.062	**0.043**
TAPSE, mm	17 ± 11	17 ± 9	0.853	16 ± 3	18 ± 2	0.253	0.974	0.947
Tissue Doppler E/E	48 ± 0	12 ± 8	–	15 ± 8	16 ± 9	0.258	**0.014**	0.541
FVC (L)	3 ± 1	3 ± 1	0.595	2 ± 1	1	(a)	0.053	0.452
FVC % pred	96 ± 18	104 ± 16	0.691	58 ± 19	57	(a)	**0.017**	0.255
FEV1, L	2 ± 0	2 ± 0	0.874	1 ± 0	1	(a)	**0.025**	0.263
FEV1, %	92 ± 28	103 ± 24	0.795	51 ± 16	38	(a)	**0.017**	0.273
FEV1/FVC, %	74 ± 11	76 ± 10	0.805	74 ± 7	56	(a)	0.976	0.350
TLC, L	5 ± 1	6 ± 1	0.772	5 ± 1	5	(a)	0.701	0.744
TLC % pred	91 ± 15	102 ± 9	0.677	90 ± 21	105	(a)	0.955	0.854
DLCO, mmol/min/kPa	5 ± 0	5 ± 1	0.670	4 ± 1	1	(a)	0.105	0.123
DLCO, %	74 ± 14	76 ± 9	0.726	51 ± 14	18	(a)	0.125	0.116
VO_2_AT, ml/min/kg	8 ± 5	6 ± 3	0.425	8 ± 2	8 ± 1	0.612	0.900	0.289
VE/VCO_2_ slope	41 ± 3	47 ± 1	0.205	41 ± 5	44 ± 10	0.600	0.960	0.777
eGFR, ml/min	35 ± 16	31 ± 9	0.594	49 ± 23	42 ± 18	0.295	0.365	0.386
AHI, events/h	24 ± 13	31 ± 0	0.674	3 ± 3	2 ± 2	0.701	0.098	** <0.001**
ODI, events/h	23 ± 14	35 ± 0	0.272	3 ± 4	3 ± 3	0.162	0.130	** <0.001**
MeanSPO_2_, %	92 ± 2	94 ± 1	0.205	94 ± 3	92 ± 3	0.342	0.236	0.634
MinSPO_2_, %	70 ± 18	81 ± 2	0.874	80 ± 11	72 ± 13	0.089	0.311	0.415
*t*90 (SaO_2_ <90% min)	157 ± 75	60 ± 49	0.137	68 ± 132	170 ± 194	0.598	0.333	0.483

*Data were presented as mean ± SD; AHI, apnea/hypopnea index; AI, apnea index; ODI, Oxygen Desaturation Index; minSPO_2_, minimal pulse oxyhemoglobin saturation; t90, oxygen saturation (SaO_2_) < 90%; mean SPO_2_, mean pulse oxyhemoglobin saturation; LVEF, left ventricular ejection fraction; RV-FAC, right ventricular fractional area change; TAPSE. tricuspid annular plane systolic excursion; BNP, B-type natriuretic peptide; eGFR, estimated glomerular filtration rate; VE/VCO2 slope, rate of increase in ventilation per unit increase in carbon dioxide; TLC, total lung capacity*.

*P, Paired sample test*.

P^*^and P^#^: Independent sample t-test;

P^*^ *means the comparisons between groups for the pre time point;*

P^#^ *means the comparisons between groups for the post time point.*

*(a) means no testing only 1 subject for the post time point of the control-SDB group; The bold values show significant difference*.

## Discussion

To the best of our knowledge, this is the first time to investigate the effect of CAVI on SDB in patients with right heart failure and severe symptomatic TR. The main finding from this investigation showed that CAVI had no obvious effect on SDB in patients with HF and TR.

Previous studies have indicated that SDB is associated with postoperative complications after general and cardiac surgery (14–16). In another study, they found SDB to be associated with a higher rate of long-term cardiovascular events after coronary artery bypass grafting (17). A study found that SDB is highly prevalent in patients undergoing cardiovascular surgery. However, in this population, the authors did not find an association between SDB and adverse postoperative outcomes due to a relatively small sample size (107 patients) (18). In our study, we also did not find any association between SDB and adverse outcome after CAVI.

SDB is known to be associated with heart disease, e.g., heart failure, coronary artery disease, and atrial fibrillation (19), but less is known about its prevalence in valve diseases. Past studies indicated a high prevalence of SDB in patients with severe aortic stenosis. Printz et al. (20) reported SDB in 15 out of 42 individuals (36%) with high-grade aortic stenosis. In our study cohort of patients with severe symptomatic tricuspid regurgitation before CAVI, the prevalence of SDB was as high as 50%. Although the total number of subjects is very small, these findings should increase the awareness of existence of SDB in patients with severe symptomatic tricuspid regurgitation.

The relation of subclinical lung function impairment with cardiovascular diseases in the absence of diagnosed pulmonary diseases has recently drawn more attention. In a cohort with long-term follow-up, low FEV1 was strongly and independently associated with incident CHF (21). A population-based study of middle-aged men observed the association between moderately reduced FEV1 and FVC and incident heart failure hospitalization (22). In our population based on relatively older subjects and limited patients sample, we demonstrated that there were no significant differences between FEV1, FEV1/FVC, and TLC after CAVI (*p* > 0.05; Table 2).

A previous study showed that an increasing VE/VCO_2_ slope was a potential negative sign (23). As presented in Table 2, the VE/VCO_2_ slope showed no obvious changes post-CAVI probably due to our very small sample size. The past study showed that the VE/VCO_2_ slope was insignificantly correlated with the AHI, and patients with CHF-SDB have hyperpnea not only during sleep but also during exercise (24). However, the correlation coefficient between the VE/VCO_2_ slope and the AHI was less than between chemosensitivity and the AHI. Chemosensitivity could not be observed, and it was speculated that the steeper VE/VCO_2_ slope was caused by augmented chemosensitivity (25). The VE/VCO_2_ slope of CHF-SDB patients increases due to increased abnormal ventilation and perfusion and physiological lung dead space.

In a mixed sample of patients with HF and mitral or aortic valve disease, they showed an improvement of SDB after heart valve surgery (26). The CAVI procedure is a recently developed method of valve replacement for use in patients with severe tricuspid regurgitation, who cannot undergo surgery or who have a high perioperative risk (27). There were no significant differences for sleep variables between pre- and post-CAVI. This could not support that SDB is another manifestation of cardiac dysfunction. Additionally, the risk for four patients experiencing severe clinical issues after CAVI in our study was 28%. Although, to date, only limited clinical data are available regarding the efficacy of transcatheter tricuspid valve intervention (TTVI), feasibility has been shown with different techniques, including annuloplasty devices (28–30) and leaflet and coaptation devices (31, 32), both in the heterotopic (CAVI; to reduce the backflow in the venous system) and the orthotopic positions. Despite the increased risk of the patients, the current report confirms the safety and feasibility of TTVI: intraprocedural mortality was 0%, 30-day mortality and periprocedural adverse events did not change, and procedural success improved significantly, from 62 to 72.8%. Clinical experience started in 2011, when CAVI was first reported for compassionate treatment of patients with severe TR using investigational self-expandable valves. Since then, compassionate clinical use has confirmed the technical feasibility of CAVI (33). Improved procedural success is likely multifactorial and related to the following: the early learning curve effect in CAVI, which is common and universal for new devices and techniques; a better understanding of TV anatomy and disease pathophysiology; and improved and more standardized intraprocedural guidance (34).

A previous study suggested that perioperative continuous positive airway pressure (CPAP) treatment could improve the AHI. Effective treatment of SDB can alleviate diastolic dysfunction (35). Whereas SDB did not improve significantly after CAVI, in this small patient cohort, we were unable to demonstrate a direct correlation between SDB improvement and CAVI procedure. The pathogenesis of SDB in HF is complex and remains to be incompletely understood. It is unclear whether SDB directly affects chronic HF pathophysiology. Therefore, the causal link to the prognosis of HF is not clear. Possibly SDB is rather an index for the severity of HF. Further studies with larger sample sizes and with pre- and post-operative evaluations are necessary.

## Study Limitations

There are many limitations of the current pilot study that need to be addressed. Our study was nonblinded and conducted about 30 days after the intervention. This is a fairly short interval for the patients to adapt to the new hemodynamic condition. Therefore, it is recommended to repeat a double-blinded study with longer periods for follow-up. The individual differences of the participants, such as their gender, age, and psychological condition, might also influence the pattern and quality of sleep. Moreover, our results are based on single-night portable monitoring; therefore, internight variations remain to be unaccounted for. Finally, this study was stopped early because of a major complication after CAVI, carried out in a single center and on a small sample of elderly people. Multicenter studies with large sample sizes are needed for improving outcomes.

## Conclusion

In summary, these data suggest that CAVI may not have an effect on SDB; however, additional follow-up fully powered studies with appropriate statistical analyses are needed.

## Data Availability Statement

The original contributions presented in the study are included in the article/supplementary material, further inquiries can be directed to the corresponding author/s.

## Ethics Statement

The studies involving human participants were reviewed and approved by Landesamt fur Gesundheit und Soziales Berlin, Germany. The patients/participants provided their written informed consent to participate in this study.

## Author Contributions

YW, RB, and RF contributed to the data collection and analysis. CS planed the study, is the guarantor of the manuscript, and assumes responsibility for the integrity of the data. TP contributed to coordinating this project. All authors contributed to drafting or revising the article, gave final approval of the version to be published, and agree to be accountable for all aspects of the work.

## Conflict of Interest

The authors declare that the research was conducted in the absence of any commercial or financial relationships that could be construed as a potential conflict of interest.
